# Applying the workload indicators of staffing needs method in determining frontline health workforce staffing for primary level facilities in Rivers state Nigeria

**DOI:** 10.1186/s41256-019-0125-z

**Published:** 2019-12-06

**Authors:** Sunny Okoroafor, Samuel Ngobua, Maritza Titus, Idonniboyeobu Opubo

**Affiliations:** 1 IntraHealth International, 30 Sudan Street, Abuja, Nigeria; 2 IntraHealth International, Eros, PO Box 9942, Windhoek, Namibia; 3 Rivers State Ministry of Health, Port Harcourt, Rivers State Nigeria

**Keywords:** Workload indicators of staffing needs, Health workforce, Human resources for health, Frontline health workers, Nurse midwives, Community health practitioners, Community health officers, Community health extension workers

## Abstract

**Background:**

Nigeria faces health workforce challenges and poor population health indices resulting from disparities in health worker densities by geographical locations and levels of health care delivery. Nigeria is constantly reforming its health system with the primary aim of having the right number of health workers in the right place at the right time to meet the population’s health needs. The majority of primary health facilities in the country are staffed using perceived needs. The Workload Indicators of Staffing Need (WISN) tool developed by the World Health Organization is used to determine staffing requirements for facilities.

**Methods:**

The WISN tool was used in assessing the staffing requirements for nurses/midwives and community health practitioners in 26 primary health facilities in Port Harcourt City Local Government Area (PHALGA) and Obio Akpor Local Government Area (OBALGA). Documents were reviewed to obtain information on working conditions and staffing, and interviews conducted with key informants in 12 randomly selected facilities. We supported an expert working group that comprised of nurses/midwives and community health practitioners to identify workload components and activity standards and validate both. We also retrieved workload data from January 1–December 31, 2015 from the national district health information system.

**Results:**

Findings showed varying degrees of shortages and inequitable distribution of health workers. Health facilities in PHALGA had a WISN ratio of 0.63 and a shortage of 31 nurses/midwives. There was also a shortage of 12 community health practitioners with a WISN ratio of 0.85. OBALGA had a shortage of 50 nurses/midwives and 24 community health practitioners; and WISN ratios of 0.60 and 0.79 for nurses/midwives and community health practitioners respectively.

**Conclusion:**

Our findings provide evidence for policies that will help Nigeria improve the population’s access to quality health services and reduce inequities in distribution of the health workforce. Evidence-based health workforce planning and redistribution using WISN should be institutionalized. Review of scopes of practice of health workforce should be conducted periodically to ensure that the scope of practice matches the training received by the specific cadres and those skills are used to deliver quality services.

## Background

Globally, access to quality health care is affected by numerous human resources for health (HRH) challenges, which are more prominent in developing countries including Nigeria. They include shortages and inequitable distribution of health workforce, poor HRH planning, uninformed policy decisions, inadequate recruitment and retention structures, and inadequate training capacities [1–4]. These result in disparities in health workforce densities by geographical locations (urban and rural areas) [5, 6] and levels of health care delivery [7]. Disparities in distribution of health workers have been strongly associated with lack of access to quality health care [5, 8] and poor health indices of populations [9].

Nigeria is constantly reforming its health system and facility staffing methods with the primary aim of having the right number of suitably-trained and equitably-distributed health workers to meet population needs. The government ministries, departments, and agencies regulating health care delivery developed facility staffing norms to ensure that the health workforce required to meet the health needs of the population is readily available. The Minimum Standards for Primary Care Delivery by the National Primary Health Care Development Agency (NPHCDA) is a document that indicates the minimum staffing norms for care at the primary health care level [10]. This level of care is critical as it is the entry point into the Nigerian health system where preventive, promotive, and curative services for uncomplicated minor ailments and referral services for complicated ailments are provided. This level of care is designed to be staffed by medical officers, nurses, midwives, community health practitioners, laboratory technicians, pharmacy technicians, health records assistants, and environmental health officers. However, the cadre of staff available at this level of care is dependent on the degree or amount of investment and commitment of the respective state and local governments [5]. Consequently, these proposed standards are domesticated by the states based on fiscal space.

Evidence shows some primary-level facilities are staffed using the NPHCDA Minimum Standards for Primary Care Delivery norm whilst a majority are not staffed using any evidence-based method nor the practitioner-to-population ratio threshold but by perceived needs [10]. As a result, the contextual variations in health seeking behaviour, service utilization rates, the daily activities conducted by health workers and the time expended in service delivery, as well as the regional discrepancies in morbidities, are not considered in staffing the facilities. In view of the numerous HRH staffing challenges, the World Health Organization developed the Workload Indicators of Staffing Need in the late 1990s.

The WISN method [11, 12] provides health care managers and planners with appropriate tool for staffing decision making; determining the number of specific type of health workers required for services at a health facility; and estimating the pressure of work on health workers at the facility. Some of its advantages are: simple to operate and uses routine health facility data; applicable to every service delivery and cadre; adaptable, acceptable and understandable even to managers without a health background. Hence, WISN findings can be used in formulating various health workforce decisions: distribution of new staff or redeployment of existing staff based on workload pressure; planning for reallocation or transfer of functions—task shifting and sharing; planning for future staffing of health care service delivery based on anticipated workloads; and examining impact of employment conditions (e.g., length of work week, vacations, in-service trainings) on staffing requirements of health facilities. The WISN method has been applied in several developing countries and evidence has shown shortages and inequitable distribution of health workforce by level of care and geographical locations. These findings have been useful in evidence-based health workforce policy development, planning, and management [13–21].

The aim of the study was to determine the staffing requirements of the frontline health workforce for select primary level facilities in Rivers State in Nigeria. This was informed by the drive of Rivers State Government to maximally utilize the current stock of frontline health workers, and improve access of the people to health workers and primary health care services towards achieving universal health coverage (UHC).

## Methods

WISN is a human resource planning tool that takes into account certain information in calculating staffing requirements for health service delivery points. The required information includes the common activities performed by a given cadre on a day-to-day basis at a specific health service delivery level (i.e., workload components), the time it takes a cadre to conduct core activities and associated activities (i.e., activity standards), the time available in 1 year for a specific cadre to execute their work (i.e., available working time [AWT] in a year) and the annual service delivery statistics in the selected health service delivery point [11, 12]. Health service activities are the core functions that all members of a cadre perform on a daily basis and for which service statistics are collected. Support activities relate to those activities performed by all members of a cadre, but for which service statistics are not being collected. Additional activities relate to those activities that are only performed by certain members of a cadre and regular statistics are not collected on them. These different workload components are then used to determine activity standards—the time a trained, well-motivated member of a particular staff category takes to perform an action to acceptable professional standards in the service delivery point. From the activity standards for health services activities and the AWT, the standard workload (amount of health service work that one member of a staff category can do in a year) is derived. Combining this with annual service statistics and activity standards for support and additional activities results in the calculated requirement for staffing at a particular health facility.

### Scope and setting

The application of the WISN methodology in Rivers State [22] was conducted between February and August 2016 in primary level health facilities, model primary health care centers (MPHCs) and primary health care centers (PHCs) in two LGAs—Port Harcourt City and Obio Akpor. The assessment was conducted by key state stakeholders from the Rivers State Ministry of Health and the Rivers State Primary Health Care Management Board and IntraHealth International with funding from the United States Agency for International Development (USAID) under the Nigeria CapacityPlus Associate Award project, led by IntraHealth. The WISN exercise focused on government-owned primary level health facilities.

### Cadres

The WISN study used workloads to ascertain the appropriate number of nurses/midwives and community health practitioners (community health officers and community health extension workers) needed to deliver quality health services to professional standards at primary level facilities in Rivers State. These cadres were selected as they are the largest providers of basic primary health care services accounting for 54% of the health workforce in this level of care and the only cadres available in most facilities [23].

### Sample technique

Purposively selected sites for this study were all PHC in operation for 1 year prior to commencement of the study in Obio Akpor and Port Harcourt City LGAs, irrespective of their staffing thresholds. In all, a total of 26 PHC (14 in Obio Akpor and 12 in Port Harcourt City) which had been functional for at least 1 year were included in the WISN exercise.

### Data collection

National and state public service rules and grey literature were reviewed to obtain information on working hours per day, working days per week, and authorized and unauthorized absences for nurses/midwives and community health practitioners in primary level health facilities in the state. Data on staffing of health facilities were also obtained from the nominal rolls (record of personnel information) of the LGAs. Data on health service workload activities (hospital statistics) for January 1, 2015 to December 31, 2015 were retrieved from the national district health information system (DHIS) 2.0 by the technical lead and a state data officer independently.

An expert working group (EWG) was formed and supported to identify the workload components and the activity standards, and to validate both during workshops. The EWG comprised representatives of the nurses/midwives and community health practitioners cadres with strong technical and practical professional experience and currently working in primary level health facilities in both rural and urban areas, pre-service and in-service training institutions for both cadres, regulatory bodies and professional associations for both cadres, and representatives of government agencies responsible for governance (Rivers State Ministry of Health and Rivers State Primary Health Care Management Board), supervision, and coordination of the selected cadres.

Information obtained from these sources was used to develop a data collection tool adapted from the data elements in the WISN software for use in validating information on staffing, working schedule of health workers (working days per week and working hours per day), authorized and unauthorized absences, and health service activities and their annual statistics. The tool was administered on purposefully selected key informants (health workers) in 12 health facilities by an interviewer following written consent from the interviewees. The data obtained was transferred into the WISN software for analysis. Information obtained from this process was used to validate information obtained from the review.

### WISN calculations

WISN requires key data inputs for calculation of staffing requirements. These include the AWT, current staffing, workload components, activity standards, and annual workload statistics.

The AWT is the time a member of a cadre has to perform his or her daily functions in a year taking into account approved and unapproved absences [11, 12]. This was calculated using information obtained from government documents and health facilities on working days per week, working hours per day, annual leave, public holidays, casual leave, compassionate leave, and estimated training days (Table 1). Information on staffing of health facilities was obtained from the nominal rolls (record of personnel information) validated against the interviews conducted at the health facility levels.

**Table 1:** Available working time (awt) for cadres of focus

CADRE	WORKING DAYS PER WEEK	WORKING HOURS PER DAY	ANNUAL LEAVE	PUBLIC HOLIDAYS	SICK LEAVE/CASUAL LEAVE	COMPASSIONATE LEAVE	TRAINING DAYS PER YEAR	AWT IN WEEKS PER YEAR	AWT IN DAYS PER YEAR	AWT IN HOURS PER YEAR
Nurse/Midwives	5	8	30	12	7	14	30	33.4	167	1336
CHO/CHEWS	5	8	30	12	7	14	30	33.4	167	1336

Workload components and activity standards are the activities that well-motivated mid-career health workers perform for the majority of their time and the time it would take them to perform the activities to professional standards within local situations. The workload components and activity standards presented in Tables 2, 3 and 4 for the health service, support activities and additional activities respectively were defined, set, and validated by EWG members during series of workshops and these were re-validated during field visits through observation [22].

**Table 2:** Validated health service acitvities and service standards for primary healthcare centre

HEALTH SERVICES	SERVICE STANDARDS FOR CADRES		UNIT TIME
	NURSE/MIDWIVES	CHOS/ CHEWS	
Antenatal Clinic (ANC)- First Visit	56	35	Minutes/patient
Antenatal Clinic (ANC)- Subsequent Visits/Revisits	31	23	Minutes/patient
Routine Immunization	23	20	Minutes/patient
Child Welfare Clinic (Sick Child) U5	20	37	Minutes/patient
Family Planning - Counselled	16	32	Minutes/patient
Family Planning - Oral	2	6	Minutes/patient
Family Planning - Injectable	6	9	Minutes/patient
Family Planning - Insertion (IUCD &Implant)	13	14	Minutes/patient
Treatment Of Minor Ailments (Children And Adults)	22	24	Minutes/patient
Delivery (Normal Delivery)	61	149	Minutes/patient
Delivery (Assisted)	80		Minutes/patient
Post Natal Care (Booked Case And Unbooked)	21	24	Minutes/patient
PMTCT - Mothers (Booked And Unbooked)	11	22	Minutes/patient
PMTCT - Infant (Booked And Unbooked)	18	34	Minutes/patient
Accidents & Emergencies - Minor Cases	27	22	Minutes/patient
Accidents & Emergencies - Major Cases	9	13	Minutes/patient
Care Of A Patient With Tuberculosis (TB)	20	20	Minutes/patient
2-Way Referrals	6	8	Minutes/patient

**Table 3:** Validated support activities and category allowance standards for primary healthcare centre

Workload components	Actual Working Time per Cadre	
	NURSES/MIDWIVES	CHO/CHEW
Community Mobilization and Education	8 h/month	12 h/month
Group Health Education	2 h/week	1 h/week
Community Development Committees (CDC) meetings	2 h/month	2 h/month
Ward Development Committees (WDC) meetings	2 h/month	2 h/month
Daily cleaning	9 mins/day	9 mins/day
Outreaches/Community-based services	8 h/month	12 h/month
Handing over/taking over, Report writing and ward round (inpatient and outpatient)	90 mins/day	90 mins/day
Follow-up care/Home visits	2 h/week	2 h/week
Staff Meetings	2 h/month	2 h/month
Documentation on patients	1 h/month	1 h/month

**Table 4:** Validated additional activities and individual allowance standard for primary healthcare centre

Workload components	NURSES/MIDWIVES		CHO/CHEW	
	Number of staff performing the task	Actual Working Time	Number of staff performing the task	Actual working Time
Supervision of students	10	32 h/month	10	2 h/week
General administration	1	3 h /month	1	3 h/month
Monthly Report writing	1	1 h/month	1	1 h/month
Review meetings (LGA coordination meeting)	1	3 h/ month	1	2 h/month
Mentoring of subordinates	1	240 min/ month	1	240 min/month
LGA Technical Meetings	1	2 h/ month	1	2 h/month
PHC Management committee meeting/Facility Management Meeting	1	2 h/ month	1	2 h/month
Advocacy	1	360 min/ year	1	600 min/year
Bed Making	2	24 min /day	2	24 min/day
Sterilization of equipment	1	30 min/day	1	30 min/day

Data on annual workload statistics of the health facilities were obtained from the national health management information system, specifically the monthly summary form version 2013. Workload data on the following health service activities were obtained: antenatal care, post-natal care, immunization, family planning, child welfare (sick child), minor ailments (children and adults), deliveries, HIV counselling and testing, prevention of mother-to-child transmission of HIV, minor and major cases of accidents and emergencies, tuberculosis, and two-way referrals.

### Data analysis and interpretation

The technical task force completed the WISN software using the state-specific information on AWT, validated workload components and activity standards, annual workload statistics, and current staffing of health facilities. Information on the WISN difference and ratio was generated and used in taking staffing decisions. The WISN difference, a difference between the current facility staffing and the calculated staff requirement, shows the level of shortage or excess of staffing to provide services. Whilst a positive value shows surplus, a negative one indicates a deficiency. The WISN ratio, calculated as current staffing divided by calculated required staffing, is used as a measure for assessing the level of workload pressure.

## Results

Tables 5 and 6 show the WISN results for nurses/midwives and community health practitioners’ cadres in Port Harcourt City LGA (PHALGA) and Obio Akpor LGA (OBALGA), respectively. The results for PHALGA as presented in Table 5 show the WISN ratio for nurses/midwives as 0.63. This indicates that PHALGA has only 63% of the required nurses/midwives. According to the WISN estimate they require 83 nurses/ midwives and have 52demonstrating a shortage of 31 nurses/midwives. These results were based on the annual workload from 12 health facilities in PHALGA. Three health facilities in this LGA have either the right number or relatively more nurses/midwives than they require while the remaining have varying degrees of shortages (1 to 8). The WISN ratio of the facilities with shortage varies from 0.29 to 0.67 indicating that these facilities have between 29 to 67% of the required nurses/midwives. The WISN results for community health practitioners in PHALGA also indicate a shortage of 12 workers with an average WISN ratio of 0.85. Seven health facilities have adequate or surplus practitioners to meet their annual workloads and five facilities with shortages have WISN ratios between 0.41 and 0.67 indicating they have 41 to 67% of community health practitioners required to meet the workload requirements. A WISN ratio of more than 1 indicates that more members of the cadre are available than what is required for the annual workload.

**Table 5:** Wisn results for nurses/midwives and community health practitioners for port harcourt city local government area (phalga)

NAME OF PRIMARY HEALTH CARE CENTER	NURSES/MIDWIVES				COMMUNITY HEALTH PRACTITIONERS			
	EXISTING STAFF	CALCULATED REQUIREMENT	GAP/EXCESS	WISN RATIO	EXISTING STAFF	CALCULATED REQUIREMENT	GAP/EXCESS	WISN RATIO
Abuloma MPHC	4	3	1	1.33	7	3	4	2.33
Amadi Ama MPHC	2	3	-1	0.67	5	2	3	2.50
Azubie MPHC	5	5	0	1.00	4	4	0	1.00
Bundu Ama MPHC	2	7	−5	0.29	2	2	0	1.00
Churchill MPHC	8	12	−4	0.67	7	17	−10	0.41
Elekahia MPHC	6	12	−6	0.50	6	6	0	1.00
Mgbundukwu MPHC	5	9	−4	0.56	10	15	−5	0.67
Mini PHC	2	4	−2	0.50	3	5	−2	0.60
Okuru-ama MPHC	9	17	−8	0.53	3	5	−2	0.60
Orogbum PHC	–	–	–	–	8	12	−4	0.67
Ozuboko MPHC	5	5	0	1.00	8	5	3	1.60
Potts Johnson MPHC	4	6	−2	0.67	6	5	1	1.20
TOTAL FOR PHALGA	52	83	−31	0.63	69	81	−12	0.85

**Table 6:** Wisn results for nurses/midwives and community health practitioners for Obio akpor local government area (obalga)

NAME OF PRIMARY HEALTH CARE CENTER	NURSES/MIDWIVES				COMMUNITY HEALTH PRACTITIONERS			
	EXISTING STAFF	CALCULATED REQUIREMENT	GAP/EXCESS	WISN RATIO	EXISTING STAFF	CALCULATED REQUIREMENT	GAP/EXCESS	WISN RATIO
Elelenwo PHC	3	4	−1	0.75	8	6	2	1.33
Eliozu MPHC	6	7	−1	0.86	4	5	−1	0.80
Eneka MPHC	6	8	−2	0.75	6	6	0	1.00
Iriebe PHC	7	10	−3	0.70	4	12	−8	0.33
Ozuoba MPHC	6	10	−4	0.60	11	14	−3	0.79
Rukpokwu MPHC	7	12	−5	0.58	3	4	−1	0.75
Rumuekini PHC	–	–	–	–	7	7	0	1.00
Rumueme MPHC	7	6	1	1.17	9	4	5	2.25
Rumuepirikom MPHC	9	9	0	1.00	10	7	3	1.43
Rumuigbo MPHC	5	10	−5	0.50	8	12	−4	0.67
Rumuodomaya MPHC	7	10	−3	0.70	6	5	1	1.20
Rumuokwrushi MPHC	5	30	−25	0.17	4	21	−17	0.19
Rumuolumeni MPHC	5	7	−2	0.71	4	7	−3	0.57
Woji MPHC	3	3	0	1.00	6	4	2	1.50
TOTAL FOR OBALGA	76	126	−50	0.60	90	114	−24	0.79

Table 6 shows the WISN results for nurses/midwives and community health practitioners in OBALGA. Overall, there is a shortage of 50 nurses/midwives and 24 community health practitioners and an average WISN ratio of 0.60 and 0.79 for nurses/midwives and community health practitioners, respectively. However, there is a lot of variation across the facilities. Rumuokwrushi MPHC has only 17% of the required nurses/midwives compared to Rumueme MPHC that has 117%. Similarly, Rumuokwrushi has only 19% of the community health practitioners it needs whereas Rumueme has 225%.

## Discussion

Our findings strengthen similar findings of shortages and inequitable distribution of the health workforce in Africa. We provide further evidence of non-availability of frontline health workers to serve local populations [13–17] especially at primary levels of care [15, 17]. While our study provides information on nurses/midwives and community health practitioners’ at primary level of care, studies on the scenario at secondary and tertiary levels are needed as these would provide further information for reviewing staffing norms and scopes of practice. Worthy of note is that the scopes of practice and titles for cadres across countries are different and this makes comparison more challenging.

The current emphasis of the national and state governments is to deliver quality basic primary care services towards the attainment of universal health coverage (UHC). The WISN method offers an evidence-based approach to calculate staffing levels needed for the delivery of quality basic primary care services considering contextual variations in health seeking behaviour, service utilization rates, the daily activities conducted by health workers and the time expended in service delivery, and the regional discrepancies in morbidities. Our findings provide evidence for several policy declarations that will help the government in achieving UHC, the Sustainable Development Goals (SDGs), and set national and state goals that will improve health indices. Government at all levels would benefit by instituting the WISN method into the health workforce planning and management policies, strategies, and plans. This will ensure that periodic evidence-based redistribution of health workers based on workload is institutionalized to further improve access to health care by individuals and quality service delivery, and to ease workforce shortages in certain facilities. It will also help in evidence-based periodic review of the minimum standards staffing norm [17] for primary health care service delivery using evidence and contribute in reducing the high internal migration of nurses/midwives to secondary and tertiary levels of care due to better remuneration packages and working conditions which is a major reason for the current low staffing levels for this cadre. These will help in ensuring that quality services are delivered by the right number of health workers, clients’ needs are met to professional standards, and the right numbers of health workers are trained to meet the current and future health workforce requirements.

A review of scopes of practice of health workforce cadres should also be conducted periodically based on the current overlap in functions at this level of care as evidenced in the identified workload components for the cadres at primary level of care [22]. The review of the scopes of practice should include plans for trainings and supportive supervision to ensure that cadres have the capacity to provide quality services [17]. This will facilitate the appropriate reallocation of tasks between cadres considering competencies, the shortages and inequitable distribution of health workforce and current workloads at service delivery points [15, 17].

Our findings informed decision by the state authorities to scale up conduct of WISN study in the state, reallocate tasks to other available cadres at health facilities and redeploy health workers based on the workload pressure. Findings also catalysed conduct of WISN study in other states in the country at a larger scale and inclusion of WISN as a national strategy for maximally using the current stock of nurses/midwives and community health workers at this level of care as a means to improving quality of care.

Our study had some limitations. There were marginal variations in the activity standards set for the cadres during workshops due to varying levels of experience among group members and the context of service delivery. In reaching a consensus on the activity standards, an average of the suggested standards was agreed upon for use. Weak documentation of health service data at facility level and DHIS, evident as non-availability of data for some months and input of non-corresponding data, prevented 100% triangulation of data obtained from the various data sources. This may have resulted in under or over estimation of staffing requirements for some health facilities. We also noticed overlapping tasks among the nurses/midwives and community health practitioners’ cadres and this posed a challenge for some workload components. This may have also impacted the under or over estimation of staffing requirements for health facilities.

## Conclusions

There is a need for government to efficiently maximize the insufficient number of health workforce taking into account health seeking behaviour patterns. WISN findings offer information for evidence-based HRH planning and for informing staffing requirements and task shifting scenarios by taking into account workloads, scope of practice, and competency of staff categories existing in the health facilities. The rigorous and procedural processes of applying the WISN method, collaboration and active participation of stakeholders at various levels, and validation of workload components and activity standards obtained from various data sources, results in reliable findings and evidence needed for policy and staffing norms.

## Data Availability

Data and materials are available on request.

## Abbreviations

AWT: Available Working Time
CAF: Category Allowance Factor
CAS: Category Allowance Standard
CHP: Community Health Practitioners
DHIS: District Health Information System
EWG: Expert Working Group
FMoH: Federal Ministry of Health
HRH: Human Resources for Health
LGA: Local Government Area
MPHC: Model Primary Healthcare Centre
NPHCDA: National Primary Health Care Development Agency
OBALGA: Obio Akpor Local Government Area
PHALGA: Port Harcourt Local Government Area
PHC: Primary Healthcare Centre
UHC: Universal Health Coverage
USAID: United States Agency for International Development
WHO: World Health Organization
WISN: Workload Indicators of Staffing Needs

## References

[1] Africa Health Workforce Observatory, World Health Organization and the European Union. Human Resources for Health Country Profile: Nigeria; 2008. http://www.who.int/workforcealliance/knowledge/resources/hrh_profile_nigeria/en/. Accessed July 10, 2016.

[2] Chen L, Evans T, Anand S, Boufford JI, Brown H, Chowdhury M, Cueto M, Dare L, Dussault G, Elzinga G, Fee E, Habte D, Hanvoravongchai P, Jacobs M, Kurowski C, Michael S, Pablos-Mendez A, Sewankambo N, Solimano G, Stilwell B, De Waal A, Wibulpolprasert S (2004). Human resources for health: overcoming the crisis. Lancet.

[3] Wyss K (2004). An approach to classifying human resources constraints to attaining health-related millennium development goals. Hum Resour Health.

[4] Wang Y, Collins C, Tang S, Martineau T (2002). Health systems decentralization and human resources management in low and middle income countries. Public Admin and Dev.

[5] Abdulraheem IS, Olapipo AR, Amodu MO (2012). Primary health care services in Nigeria: critical issues and strategies for enhancing the use by the rural communities. J Public Health Epidemiol.

[6] Daviaud E, Chopra M (2008). How much is not enough? Human resources requirements for primary health care: a case study from South Africa. Bull World Health Organ.

[7] Willcox ML, Peersman W, Daou P, Diakité C, Bajunirwe F, Mubangizi V, Mahmoud EH, Moosa S, Phaladze N, Nkomazana O, Khogali M, Diallo D, De Maeseneer J, Mant D (2015). Human resources for primary health care in sub-Saharan Africa: progress or stagnation?. Human Resour for Health.

[8] Aluttis C, Bishaw T, Frank MF (2014). The workforce for health in a globalized context-global shortages and international migration. Glob Health Action.

[9] World Health Organization (2006). The world health report 2006: working together for health.

[10] National Primary Health Care Development Agency. Minimum Standards for Primary Health Care in Nigeria. http://www.nphcda.gov.ng/Reports%20and%20Publications/Minimum%20Standards%20for%20Primary%20Health%20Care%20in%20Nigeria.pdf. (accessed August 20, 2016).

[11] Shipp PJ (1998). Workload indicators of staffing need (WISN): a manual for implementation.

[12] World Health Organization (2010). Workload indicators of Staffing Need. User’s manual.

[13] Hagopian A, Mohanty MK, Das A, House PJ (2012). Applying WHO’s ‘workforce indicators of staffing need’ (WISN) method to calculate the health worker requirements for India’s maternal and child health service guarantees in Orissa state. Health Policy Plan.

[14] Kolehmainen-Aitken RL, Shipp P (1990). ‘Indicators of staffing need’: assessing health staffing and equity in Papua New Guinea. Health Policy Plan.

[15] McQuide PA, Kolehmainen-Aitken RL, Forster N (2013). Applying the workload indicators of staffing need (WISN) method in Namibia: challenges and implications for human resources for health policy. Hum Resour Health.

[16] Musau P, Nyongesa P, Shikhule A, Birech E, Kirui D, Njenga M, Mbiti D, Bett A, Lagat L, Kiilu K (2008). Workload indicators of staffing need method in determining optimal staffing levels at Moi teaching and referral hospital. East Afr Med J.

[17] Namaganda G, Oketcho V, Maniple E, Viadro C (2015). Making the transition to workload-based staffing: using the workload indicators of staffing need method in Uganda. Human Resour Health.

[18] World Health Organization (2010). Applying the WISN Method in practice: case Studies from Indonesia, Mozambique and Uganda.

[19] World Health Organization (2016). Workload Indicators of Staffing Need (WISN): selected country implementation experiences. Human Resources for Health Observer Series No. 15.

[20] Govule P, Mugisha JF, Katongole SP, Maniple E, Nanyingi M (2015). Onzima Robert Anguyo D D M. application of workload indicators of staffing needs (WISN) in determining health workers’ requirements for Mityana general hospital, Uganda. Int J Public Health Res.

[21] Ly A, Kouanda S, Ridde V (2014). Nursing and midwife staffing needs in maternity wards in Burkina Faso referral hospitals. Human Resour Health.

[22] Okoroafor S, Ngobua S, Titus M, Idoniboyeobu O (2016). Determining frontline health workforce staffing for primary level facilities in Rivers state: application of the workload indicators of staffing needs (WISN) method.

[23] Rivers State Ministry of Health (2015). Human resources for health situation analysis.

